# PedGenie: meta genetic association testing in mixed family and case-control designs

**DOI:** 10.1186/1471-2105-8-448

**Published:** 2007-11-15

**Authors:** Karen Curtin, Jathine Wong, Kristina Allen-Brady, Nicola J Camp

**Affiliations:** 1 Department of Biomedical Informatics, University of Utah, Salt Lake City, UT 84108, USA

## Abstract

**Background-:**

PedGenie software, introduced in 2006, includes genetic association testing of cases and controls that may be independent or related (nuclear families or extended pedigrees) or mixtures thereof using Monte Carlo significance testing. Our aim is to demonstrate that PedGenie, a unique and flexible analysis tool freely available in Genie 2.4 software, is significantly enhanced by incorporating meta statistics for detecting genetic association with disease using data across multiple study groups.

**Methods-:**

Meta statistics (chi-squared tests, odds ratios, and confidence intervals) were calculated using formal Cochran-Mantel-Haenszel techniques. Simulated data from unrelated individuals and individuals in families were used to illustrate meta tests and their empirically-derived p-values and confidence intervals are accurate, precise, and for independent designs match those provided by standard statistical software.

**Results-:**

PedGenie yields accurate Monte Carlo p-values for meta analysis of data across multiple studies, based on validation testing using pedigree, nuclear family, and case-control data simulated under both the null and alternative hypotheses of a genotype-phenotype association.

**Conclusion-:**

PedGenie allows valid combined analysis of data from mixtures of pedigree-based and case-control resources. Added meta capabilities provide new avenues for association analysis, including pedigree resources from large consortia and multi-center studies.

## Background

In the study of common diseases and genes with modest effects, large consortium and multi-center efforts hold the promise of increased power to detect associations, but present analysis challenges. The study populations for the multiple centers may differ geographically and ethnically, and considerable differences in case-control ascertainment and pedigree structures between studies are likely. PedGenie, available as an analysis option in Genie 2.4 software, requires Java 1.6 for execution and extends the functionality previously available in PedGenie 1.2 [1] by incorporating meta statistics for combined analysis of multi-study resources, along with Monte Carlo significance testing which allows for a mixture of pedigree members and independent individuals [2]. PedGenie offers a novel, structured approach that allows valid meta association testing, where each study considered can be a mixture of family-based individuals (including pedigrees of arbitrary size and complexity) and singletons, not simply pooling of data across studies. Briefly, study-specific allele frequencies for markers of interest are used within studies. A Mendelian gene-drop is performed in each study separately, in accordance with its own allelic characteristics, independent of trait. Each possible null genotype configuration for all studies is used to create a null meta statistic, which creates an empirical null distribution for the significance testing when repeated multiple times. These methods, detailed below, have been previously described for single studies [2] and applied to existing study data in candidate gene association and transmission testing [3].

In addition to offering all the capabilities of version 1.2, PedGenie (version 2.4) includes allele and genotype association analysis across studies based on formal Cochran-Mantel-Haenszel (CMH) techniques to calculate the following meta statistics: chi-squared test of independence, chi-squared test of trend, and odds ratios and confidence intervals. An illustration of a meta-association analysis as implemented in PedGenie can be found in Curtin et al [4]. Here we formally introduce and validate the addition of these meta association statistics by using simulated data for independent individuals to illustrate that the meta tests performed in PedGenie and their associated empirically-derived p-values and confidence intervals, are accurate, precise, and match those provided by standard statistical software. Further, we illustrate the flexibility of the method with an application with simulated data of mixed study design- a design that no other software currently available can address for meta association. Simulated case-control (independent) and family-based (nuclear and extended pedigree) study data under both a null hypothesis of no association and an alternate hypothesis of a genotype-phenotype association were used.

## Implementation

PedGenie is a flexible, easily implemented, and freely available analysis tool that is enhanced significantly in Genie 2.4 software [5] by the incorporation of meta statistics to allow valid combined analysis of multiple studies in the detection of genetic association with common disease. Java 1.6 is required for execution.

## Methods

### Meta statistics

In epidemiologic studies, data are often collected that can be summarized in three-way contingency tables, the presence or absence of a disease phenotype cross-classified with allele or genotype, and controlling for a third categorical variable or 'group' [6]. In a multi-center collaboration, this third factor represents study site. Meta statistics for a genotypic or an allelic association across studies, currently incorporated in PedGenie, are based on a multivariate extension of the Cochran-Mantel-Haenszel (CMH) test to sets of (s × r) contingency tables, indexed by i = 1, 2,..., s and j = 1, 2,..., r in terms of the corresponding multiple hypergeometric model as formulated in Landis and Koch [7]. The CMH test is based on a fixed-effects approach [8], in which PedGenie assumes the same genetic effect size across studies. The generalized CMH approach was used to calculate a chi-squared general association test of independence and chi-squared test of trend (mean score statistic where ordered wildtype, heterozygous, and homozygous variant genotypes lie on an ordinal scale) [5]. Meta odds ratios in PedGenie were calculated as the Mantel-Haenszel estimate of the common odds ratio for stratified 2 × 2 tables described in [9,10], with corresponding 95% confidence limits estimated empirically, as the 2.5^th ^and 97.5^th ^percentile of odds ratios from the null genotype configurations.

An outline of steps for meta statistics in PedGenie follows. First, allele frequencies for the markers of interest are estimated from the data. This is performed separately for each study. As before, these can be estimated by four different methods: genotyped founders only, all genotyped individuals, all founders with statistically inferred genotypes, and user-defined (for details of each, see [2]). Second, alleles are assigned to the pedigree founders randomly, but in proportion to the study-specific allele frequencies, and a Mendelian gene-drop is performed. That is, the gene-drop is performed independently of trait information. The resulting null genotype configuration across all pedigrees and singletons, therefore represents a possible configuration under the null hypothesis of no association between allele and disease, maintaining study-specific allele frequencies. Third, the meta statistic of interest is calculated using the null genotype configuration and the true phenotype data, maintaining the study identities but ignoring the pedigree relationships that exist, which we term M_*i*_. The M_*i *_meta statistic is from the null distribution since it was derived from data simulated under the null hypothesis. To match the information content of the real data, we limit calculation of the statistic of interest in the simulated data to only those individuals with genotype data in the observed sample. In this way, the missing data structure is captured. Steps 2 and 3 are repeated multiple (N) times, and the series of null meta statistics stored. Hence an empirical null distribution is created for the meta statistic of interest, conditional on the particular pedigree and phenotype structures contained across all studies in the total resource.

Finally, the observed statistic, M_*0*_, is computed based on the true genotype and phenotype data using the same meta statistic of interest, again ignoring any pedigree relationships that exist. This observed meta statistic is then compared to the empirical null distribution to determine significance as follows:

p=Σ[I(Mi)]/Nfor i=1 to Nwhere:I(Mi)=1if Mi≥M00otherwise
 MathType@MTEF@5@5@+=feaafiart1ev1aaatCvAUfKttLearuWrP9MDH5MBPbIqV92AaeXatLxBI9gBaebbnrfifHhDYfgasaacPC6xNi=xI8qiVKYPFjYdHaVhbbf9v8qqaqFr0xc9vqFj0dXdbba91qpepeI8k8fiI+fsY=rqGqVepae9pg0db9vqaiVgFr0xfr=xfr=xc9adbaqaaeGacaGaaiaabeqaaeqabiWaaaGcbaqbaeaabmGaaaqaaiabdchaWjabg2da9iabfo6atjabcUfaBHqaaiab=LeajjabcIcaOiab=1eannaaBaaaleaacqWGPbqAaeqaaOGaeiykaKIaeiyxa0Laei4la8Iae8Nta4eabaGae8NzayMae83Ba8Mae8NCaiNaeeiiaaIaemyAaKMaeyypa0JaeGymaeJaeeiiaaIae8hDaqNae83Ba8MaeeiiaaIae8Nta4eabaGae83DaCNae8hAaGMae8xzauMae8NCaiNae8xzauMaeiOoaOJae8xsaKKaeiikaGIae8xta00aaSbaaSqaaiabdMgaPbqabaGccqGGPaqkcqGH9aqpcqaIXaqmaeaacqWFPbqAcqWFMbGzcqqGGaaicqWFnbqtdaWgaaWcbaGaemyAaKgabeaakiabgwMiZkab=1eannaaBaaaleaacqaIWaamaeqaaaGcbaGaeGimaadabaGae83Ba8Mae8hDaqNae8hAaGMae8xzauMae8NCaiNae83DaCNae8xAaKMae83CamNae8xzaugaaaaa@6E94@

The specified null hypothesis is rejected if the *p*-value is less than or equal to the required level of type I error (α).

### Monte Carlo p-value and confidence interval assessment

To evaluate the accuracy of the meta association statistics available in PedGenie (CMH chi-square, CMH trend, meta ORs) and corresponding empirical p-values and 95% confidence intervals (95% CI), two simulated meta-datasets were constructed, each comprised of three studies, as described below. Briefly, two test set designs were used. The first 'independent' design was comprised of three separate studies of varying sample size, each being the standard association design of independent cases and 1:1 frequency matched controls. Results from this design should be in exact agreement with results from standard statistical packages for test statistics, and in asymptotic agreement for their associated p-values and confidence intervals. The second 'mixed' design was comprised of mixtures of study designs (independent and family-based), both within and between studies. This 'mixed' design is used to illustrate PedGenie's versatility.

Within each of the two meta-analytic datasets (independent or mixed design), a variety of sample sizes and minor allele frequencies (MAFs) for one bi-allelic single nucleotide polymorphism (SNP) were used across the component studies as detailed in the following section. In each component study, a dataset was simulated under two scenarios: one under the null hypothesis of no association, and another under the alternative hypothesis of association of an allele of interest and phenotype. Under the null scenario, a sporadic rate of 0.10 for affected phenotype status was randomly assigned, regardless of genotype. Under the alternate scenario, penetrances for each genotype (AA, Aa, and aa, where A is the common allele) were determined based on a multiplicative genetic model with a genotypic relative risk (GRR) of 1.5 and a sporadic rate of 0.10, according to the methodology described in Camp [11].

Since PedGenie is an empirical approach, we repeated this procedure 1,000 times for each test design (independent or mixed) and scenario (null or alternative) by randomly re-seeding the initial assignment of alleles in each analysis to demonstrates the reproducibility of the Monte Carlo method, as equivalent results are obtained if a different random number seed is used. The means and standard deviations of the 1,000 empirical p-values and confidence intervals for each meta study simulation were examined for stability and accuracy. For the 'independent' test design, the meta statistics and p-values were compared to statistics and p-values from a standard distribution using an established statistical software package (SAS^® ^version 9.1). For each PedGenie analysis, either 1,000 configurations (for the null scenario) or 10,000 configurations (for the alternate scenario) were used to determine the empirical null distribution and p-values and confidence intervals for meta (across studies) and within-study test statistics. Generally, the number of gene-dropping configurations was guided by the expected size and desired precision of a p-value. Clearly, the accuracy of the empirical p-value increases with the size of the empirical null distribution simulated; for example, an N of 2,000 gives a 95% confidence interval around α = 0.05 with a width of 0.02 under the null hypothesis.

### Methods to generate meta-analytic datasets

#### Independent design

Three separate studies of independent cases and randomly selected, frequency-matched controls (1:1 ratio) were simulated with the following sample sizes and MAFs: 1,000 independent cases and 1,000 random controls, MAF = 0.05 (study 1.1); 500 independent cases and 500 random controls, MAF = 0.10 (study 1.2); and 250 independent cases and 250 random controls, MAF = 0.20 (study 1.3). Data were constructed for this design under both the null and alternative scenarios as described above.

#### Mixed design

Three separate studies with a mixture of both family-based and independent resources were simulated with the following sample sizes and MAFs: 1,000 independent cases and 1,000 random controls, MAF = 0.05 (study 2.1, which is equivalent to study 1.1); nuclear families consisting of 200 affected sib-pairs plus parents, 200 discordant sib-pairs plus parents, and 200 independent controls, MAF = 0.10 (study 2.2); and four-generation pedigrees consisting of 53 family members per pedigree (sibships of size three in all generations, and all sibs marry in generations 2 and 3); and including 500 independent controls, MAF = 0.20 (study 2.3).

Study characteristics resulting from the independent and mixed design simulations under the null and alternative scenarios are shown in Tables 1 and 2, respectively. To construct case-control studies 1.1 (also called 2.1), 1.2, and 1.3, large "source" populations were simulated. First, alleles were assigned to independent individuals in three "populations" of over 10,000 individuals based on the specified MAF for each study. Second, phenotype status was assigned to individuals randomly, but according to the appropriate penetrances under the either the null or alternate scenario. Third, the required number of cases and controls were selected randomly from the appropriate simulated "source" population.

**Table 1:** Simulated study characteristics, null scenario.

Study	Description	Pop. MAF	Sample size (N)			Study cases				Study controls			
			
			Total	Cases	Ctrls.	MAF	AA	Aa	aa	MAF	AA	Aa	aa
Independent design meta-analytic datasets													

1.1	Case-control	0.05	2000	1000	1000	0.06	892	105	3	0.05	898	100	2
1.2	Case-control	0.10	1000	500	500	0.10	404	91	5	0.10	409	86	5
1.3	Case-control	0.20	500	250	250	0.19	162	79	9	0.21	156	84	10

Mixed design meta-analytic datasets													

2.1	Case-control	0.05	2000	1000	1000	0.06	892	105	3	0.05	898	100	2
2.2	Nuclear families and independents^1^	0.10	1800	675	1125	0.10	546	120	9	0.10	915	196	14
2.3	Pedigrees and independents^2^	0.20	4550	501	4049	0.19	333	147	21	0.20	2584	1279	186

^1^400 affected sibs in affected sib-pairs (ASPs), 200 affected sibs in discordant sib-pairs (DSPs), and 75 family cases; 200 unaffected sibs in DSPs, 725 family, and 200 independent controls.

^2^501 family cases and 3,549 family controls in 90 four-generation pedigrees (founders and first generation ungenotyped), 500 independent controls.

**Table 2:** Simulated study characteristics, alternate scenario.

Study	Description	Pop. MAF	Sample size (N)			Study cases				Study controls			
			
			Total	Cases	Ctrls.	MAF	AA	Aa	aa	MAF	AA	Aa	aa
Independent design meta-analytic datasets													

1.1	Case-control	0.05	2000	1000	1000	0.07	865	130	5	0.05	906	92	2
1.2	Case-control	0.10	1000	500	500	0.13	375	116	9	0.09	413	83	4
1.3	Case-control	0.20	500	250	250	0.26	136	96	18	0.19	163	80	7

Mixed design meta-analytic datasets													

2.1	Case-control	0.05	2000	1000	1000	0.07	865	130	5	0.05	906	92	2
2.2	Nuclear families and independents^1^	0.10	1800	694	1106	0.16	496	180	18	0.12	853	242	11
2.3	Pedigrees and independents^2^	0.20	4190	503	3687	0.23	294	184	25	0.19	2386	1184	117

^1^400 affected sibs in ASPs, 200 affected sibs in DSPs, and 94 family cases; 200 unaffected sibs in DSPs, 706 family, and 200 independent controls.

^2^503 family cases and 3,187 family controls in 83 four-generation pedigrees (founders and first generation ungenotyped), 500 independent controls.

For the nuclear family study (study 2.2), alleles were first randomly assigned to parents in families with two siblings in a "population" of over 15,000 families (four individuals per pedigree) based on the specified MAF. Second, alleles were assigned to siblings according to Mendelian inheritance. Third, phenotype was assigned to all individuals randomly, but according to penetrances under either the null or alternate scenario. Finally, 400 nuclear families (200 affected and 200 discordant sib-pairs and their parents) were selected randomly from the "source" population. In addition, 200 independent controls were randomly selected from case-control study 1.2 from the appropriate scenario (null or alternate) to create study 2.2, a mixed family and independent resource. Genotypes on all parents, affected and unaffected, were also included in the simulation to demonstrate the flexibility of PedGenie to handle any pedigree structure and use all information available in each pedigree. This resulted in additional family-based cases and controls as noted in Tables 1 and 2.

Similarly, in extended family study (study 2.3), the initial step consisted of randomly assigning alleles to all founders in a "population" of 150 four-generation pedigrees (14 founders and 53 members per pedigree) based on the MAF specified previously. Second, a Mendelian gene-drop was performed in which alleles were assigned to offspring randomly based on parental alleles. Third, phenotype was assigned to individuals randomly, but according to penetrances under the either the null or alternate scenario. Genotype data for generations 1 and 2 were removed, such that genotypes were only considered available for the bottom two generations. Finally, pedigrees containing at least four related, genotyped cases were randomly selected until ~500 affected family members had been ascertained. This resulted in 90 pedigrees in the null scenario, and 83 pedigrees in the alternate scenario containing 501 and 503 family-based cases, respectively. In addition, 500 unaffected independent controls were randomly selected from a source "population" corresponding to the null or alternate scenario samples in case-control Study 1.3 to create a mixed pedigree and independent resource. All family members with genotypes, both affected and unaffected, were included in the simulation to demonstrate the flexibility of PedGenie to handle large pedigree structures and use all information available in each pedigree. This flexibility resulted in additional pedigree-based controls as detailed in Tables 1 and 2.

## Results

The Monte Carlo p-value and confidence interval assessment results, for 1,000 randomly seeded runs using the multi-study, independent-design meta-analytic datasets under both null and alternate scenarios, are shown in Tables 3 and 4, respectively. Likewise, the p-value and confidence interval assessment results, for 1,000 randomly seeded runs using the mixed-design meta-analytic datasets (null and alternate scenarios), are shown in Tables 5 and 6, respectively. In each run, repeated 1,000 times using a different random number seed, statistics and empirical p-values or 95% confidence intervals (CIs) were derived according to the methods in PedGenie from 1,000 null distribution configurations (null scenario), and 10,000 null configurations (alternate scenario). The statistics, mean (and standard deviation) for p-values and 95% CIs were compared to the corresponding meta statistics, standard p-values, and standard 95% CIs from established statistical software for the independent design (Tables 3 and 4).

**Table 3:** Independent design meta analysis of case-control studies, ^1 ^null scenario. ^2^

Description	CMH Chi Square	CMH Chi-Square Trend	Meta Odds Ratio	
			
			Aa vs. AA	aa vs. AA
Statistic, PedGenie 2.1	0.06	0.05	1.02	0.99
Empirical p-value, mean (SD)	0.970 (0.005)	0.829 (0.012)	0.818 (0.031)	0.967 (0.028)
Empirical 95% CI: lower (SD)			0.85 (0.007)	0.49 (0.016)
Empirical 95% CI: upper (SD)			1.24 (0.010)	2.00 (0.068)
Statistic, SAS^®^	0.06	0.05	1.02	0.99
Standard p-value	0.972	0.830	0.816	0.971
Standard 95% CI: lower			0.85	0.50
Standard 95% CI: upper			1.23	1.96

^1^Studies 1.1, 1.2, and 1.3.

^2^1,000 configurations in the PedGenie null.

**Table 4:** Independent design meta analysis of case-control studies, ^1 ^alternate scenario. ^2^

Description	CMH Chi Square	CMH Chi-Square Trend	Meta Odds Ratio	
			
			Aa vs. AA	aa vs. AA
Statistic, PedGenie 2.1	26.49	26.17	1.49	2.81
Empirical p-value, mean (SD)	<0.0001 (<0.0001)	<0.0001 (<0.0001)	<0.0001 (<0.0001)	0.0020 (0.0006)
Empirical 95% CI: lower (SD)			1.24(0.0031)	1.51 (0.0136)
Empirical 95% CI: upper (SD)			1.79 (0.0044)	5.24 (0.0475)
Statistic, SAS^®^	26.49	26.17	1.49	2.81
Standard p-value	<0.0001	<0.0001	<0.0001	0.0014
Standard 95% CI: lower			1.24	1.45
Standard 95% CI: upper			1.79	5.42

^1^Studies 1.1, 1.2, and 1.3.

^2^10,000 configurations in the PedGenie null.

**Table 5:** Mixed design meta analysis of case-control and family-based/mixed-resource studies, ^1 ^null scenario. ^2^

Description	CMH Chi Square	CMH Chi-Square Trend	Meta Odds Ratio	
			
			Aa vs. AA	aa vs. AA
Statistic, PedGenie 2.1	0.23	0.22	0.97	0.94
Empirical p-value, mean (SD)	0.886 (0.010)	0.616 (0.016)	0.630 (0.029)	0.786 (0.030)
Empirical 95% CI: lower (SD)			0.85 (0.005)	0.60 (0.013)
Empirical 95% CI: upper (SD)			1.10 (0.006)	1.48 (0.032)
Statistic, SAS^®^	0.23	0.22	0.97	0.94

^1^Studies 2.1, 2.2, and 2.3.

^2^1,000 configurations in the PedGenie null.

^3^P-values and 95% CIs are not given as SAS cannot account for dependent relationships, and hence are not appropriate.

**Table 6:** Mixed design meta analysis of case-control and family-based/mixed-resource studies, ^1 ^alternate scenario. ^2^

Description	CMH Chi Square	CMH Chi-Square Trend	Meta Odds Ratio	
			
			Aa vs. AA	aa vs. AA
Statistic, PedGenie 2.1	28.22	27.22	1.31	2.03
Empirical p-value, mean (SD)	<0.0001 (<0.0001)	<0.0001 (<0.0001)	<0.0001 (<0.0001)	0.0001 (0.0001)
Empirical 95% CI: lower (SD)			1.16 (0.0020)	1.42 (0.0069)
Empirical 95% CI: upper (SD)			1.49 (0.0026)	2.92 (0.0143)
Statistic, SAS^®^	28.22	27.22	1.31	2.03

^1^Studies 2.1, 2.2, and 2.3.

^2^10,000 configurations in the PedGenie null.

^3^P-values and 95% CIs are not given as SAS cannot account for dependent relationships, and hence are not appropriate.

For the independent design, all statistics were shown to be equivalent to those from a standard package (Tables 3 and 4). The p-values from 1,000 randomly seeded runs of PedGenie were tightly and normally distributed based on their mean and standard deviation. The corresponding median p-values and interquartile ranges were in very close agreement (data not shown). In the alternate scenario, where p-values were much smaller than in the null scenario, the majority of p-values were within 0.0001 of the value from the standard distribution (Table 4), and none were significantly different from expected based on the standard distribution.

The PedGenie software implements a Monte Carlo approach that corrects the null distribution, and not the observed statistic, for family relationships and hence the statistics for the mixed design are also the standard statistics. As shown in Tables 5 and 6, these match precisely. However the p-values and 95% CIs for the mixed design incorporate the relatedness of individuals and thus cannot be compared to results from standard statistical packages.

To illustrate performance, runtime is reported. Runtime for PedGenie (null scenario) with 1,000 null configurations took approximately 1 minute, 30 seconds per single independent-design meta analysis and under 4 minutes per single mixed-design meta analysis on a Dell^® ^Precision 670 computer with 4 gigabytes of memory, using a Red Hat^® ^linux operating system. Runtime (alternate scenario) with 10,000 null configurations took approximately 15 minutes per independent-design meta analysis and ~35 minutes per mixed-design meta analysis.

## Discussion

For common traits involving small effect genes, consortia and multi-center efforts are becoming commonplace. For example, CMH statistics for ordered genotypes have been used to assess association in multiple case-control studies in which cases are independent, either as probands or as randomly selected affecteds [12]. Complex statistical issues arise when study designs vary across groups who wish to combine data; currently, only a limited number of approaches exist to address this. A combined odds ratio/relative risk estimate of association in both case-control and transmission-disequilibrium test (TDT) studies has been proposed [13,14]. Strategies for combining evidence in case-control and nuclear families have also been developed [15-17] and one package, Pseudomarker, can also include extended pedigrees [18]. However, one or more limitations exist in these other software: only single point analyses are available; limited pedigree structures can be incorporated; and/or there exists an implicit assumption that the component studies of the mixed designs are ascertained from the same source population and are therefore not robust to marker allele frequency differences across studies. PedGenie allows for valid meta analyses of combined family-based and case-control data, both within and between studies, using CMH techniques. The tests are robust to marker allele frequency difference across studies because separate gene-drops using study-specific frequencies are used in the Monte Carlo simulations. PedGenie can accommodate comprehensive information in large and complex multi-generational families without pedigree splitting or dropping of individuals required in other packages [19-21], with acceptable processing times.

PedGenie can incorporate all relationships in family-based resources, therefore all family members with phenotype and genotype data that are available can be included. For example, if only one affected sibling was chosen from each pedigree as some investigators are forced to do to appropriately utilize standard statistical packages, the sample size in study 2.2 would have been reduced under the alternate scenario from 694 cases, 200 independent controls, and 706 family-based controls to 400, 200, and zero, respectively. This may be particularly useful for linkage consortia interested in pursuing association testing, as PedGenie can provide valid meta analyses across multiple studies each containing family-based cases and family-based controls, in addition to any supplemental independent controls available. The ability to more fully utilize all data on hand both increases the utility of prior linkage resources and provides increased power to detect associations, particularly in stratified and subset analyses that likely lead to small sample sizes in individual studies. We anticipate that the new meta capabilities in PedGenie will provide new avenues for linkage consortia to explore in terms of association testing.

A potential limitation of PedGenie meta analysis is the fixed-effects nature of the CMH approach, and interpretation of the meta odds ratio is dependent on an assumption of homogeneity in genetic effects across studies. Future work will include providing an option to test for heterogeneity across studies such as described in [8,22,23], and developing a random-effects model extension allowing for heterogeneity such as that recently proposed by Bagos and Nikolopoulos based on logistic regression [24]. When heterogeneity is not extreme, it has been shown that fixed- and random-effects models yield similar results [8]. However, use of a random-effects model does not control or adjust for heterogeneity, and choice of a fixed- or random-effects model should be secondary to examination of factors contributing to heterogeneity [8].

In this study, we have described allele and genotype tests for single SNPs. PedGenie also incorporates haplotype and composite genotype tests; these are based on the most likely haplotype configurations from expectation-maximization (both in the observed and all simulated data) rather than a full likelihood approach based on all possible haplotypes (which is not a feasible approach for pedigrees of arbitrary size). We are currently working towards more sophisticated haplotype methods. Future efforts will also include meta extensions for other quantitative and transmission statistics, such as the TDT test, and multiple logistic regression analysis (including a random-effects extension) with covariates.

## Conclusion

We have demonstrated PedGenie is a valid statistical software tool that offers a comprehensive way of performing valid meta association testing across studies of mixed design. PedGenie (freely available in Genie 2.4 software) can accommodate independent and family-based resources, allowing for more power to detect associations by allowing for valid combined analyses and maximal use of available data.

## Availability and Requirements

PedGenie is an analysis option in Genie 2.4 software, University of Utah Genetic Epidemiology Division, freely available at [5]. Java 1.6 is required for execution, along with the following files: Genie.jar (downloaded in Genie.zip), a parameter file (user-defined .rgen, XML file; go to ".rgen Parameter File" link from the Genie home page for detailed description and examples), and user-provided genotype data file(s) in pedigree format (there is no restriction on the number of files). For a description of functionality and instructions for execution, see "PedGenie" page; select "PedGenie Examples" and "Meta/CMH Analysis Example" for examples of meta statistical analysis input and output files.

## Authors' contributions

KC carried out the validation testing and statistical analysis, participated in software development, and drafted the manuscript. JW performed the Java programming and maintains the software home page. KAB participated in the design of the study and helped to draft the manuscript. NJC conceived of the study, and participated in its design and coordination and helped to draft the manuscript. All authors read and approved the final manuscript.

## Supplementary Material

Additional file 1 genie. A compressed archive file of Genie 2.4.2, including Genie.jar, genepi.jar, and all PedGenie example files. Click here for file

Additional file 2 geniesource. A compressed archive file with Genie 2.4.2 source code only. Click here for file
